# Supplementary material for: Transcriptomic Analysis Reveals the Detoxification Mechanism of Chilo suppressalis in Response to the Novel Pesticide Cyproflanilide
Source: Int J Mol Sci. 2023 Mar 13;24(6):5461. doi: 10.3390/ijms24065461 (PMC10049496; doi:10.3390/ijms24065461)
Supplement: Supplementary file 1 [file ijms-24-05461-s001.zip › ijms-2242602-supplementary.pdf]

Table S1 Summary of the transcriptome sequence data from the control and cyproflanilide treatment samples

Samples	Clen Read Number	Clen Base number	GC(%)	Q20(%)	Q30(%)
Acetone 1	43558658	6.53G	46.4	97.66	93.4
Acetone 2	39637326	5.95G	46.46	97.6	93.22
Acetone 3	40378420	6.06G	46.79	97.58	93.23
Cyproflanilide (LD_{30})-1	42770672	6.42G	46.83	97.66	93.41
Cyproflanilide (LD_{30})-2	38996026	5.85G	46.79	97.63	93.33
Cyproflanilide (LD_{30})-3	38450956	5.77G	46.29	97.61	93.26

Table S2 Primer used qRT-PCR

Unigene name	Description(blast)	Primer
Csup013766	*ABCC3*	F:CCTCAAACCGATGCCCTCAT<br>R:CTTGACCCTGGTCCATCACC
Csup013576	*ABCC6*	F:GCGTCCACGAGTTTGCATTT<br>R:TGCCACTAACCATTTTGACAGT
novel.2042	*CYP321F1*	F:TGCTGTAGGTTCAACCCCTG<br>R:AGCGGTGTGATGAAACACGA
Csup010730	*CYP6AB49*	F:CCGTGCGTCTATACCTGGTT<br>R:TGTCTGGATGAAGTCCACGC
Csup001366	*CYP4AU10*	F:CGGTGTTTGGTCAACAAGCC<br>R:TTTCTCACCCATGGCGGTTT
Csup006357	*CYP367A9*	F:CCAACGCCTTTGTACCGTTC<br>R:GGCCCCTCGGATTCTATGTG
Csup009740	*CYP4G90*	F:AGCTACGCGGCAGAAAATCT<br>R:CTGGACATCCTCCAGTACGC
Csup004590	*CYP6AB48*	F:TCTTACCCTTCGGCACTGGA<br>R:GCCGAACCGTAAACCGTGAT
Csup005874	*Csu Est5*	F:TGCAGAGCGAAGCTACATCC<br>R:AGCTTCTCCCTTTGGATCGC
Csup012348	*Csu Est17*	F:GGAGTTTGGACAAGGCGAGT<br>R: AACTCGATTCCGGCGTCTTC
Csup007894	*UGT33AG1*	F:CCACGCCGTTTTGTCATTCA<br>R:GTTCCAACCATTCCAACGGG
novel.1375	*UGT33AG3*	F:GTGTTCTCCAAACTCCCATACG<br>R:GGCCCGGCAATTCATCT
house-keeping gene	*EF-1*	F:TGAACCCCCATACAGCGAATCC<br>R:TCTCCGTGCCAACCAGAAATAGG

Table S3 Primer for RNA silence fragment synthesis

Unigene name	Description(blast)	PRC production length	Primer
Csup009740	*CYP4G90*	528bp	F:ctcccggccgccatggcggccgcCTCATCGCCCCCACTTTCC<br>R:ggataacaattcccctctagaGACATCGTTGTCGTCAACATCTAAA
Csup001366	*CYP4AU10*	553bp	F:ctcccggccgccatggcggccgcGGTGTTAGCCCTTATATTCGTATGTG<br>R: ggataacaattcccctctagaACCCATGGCGGTTTCGAA

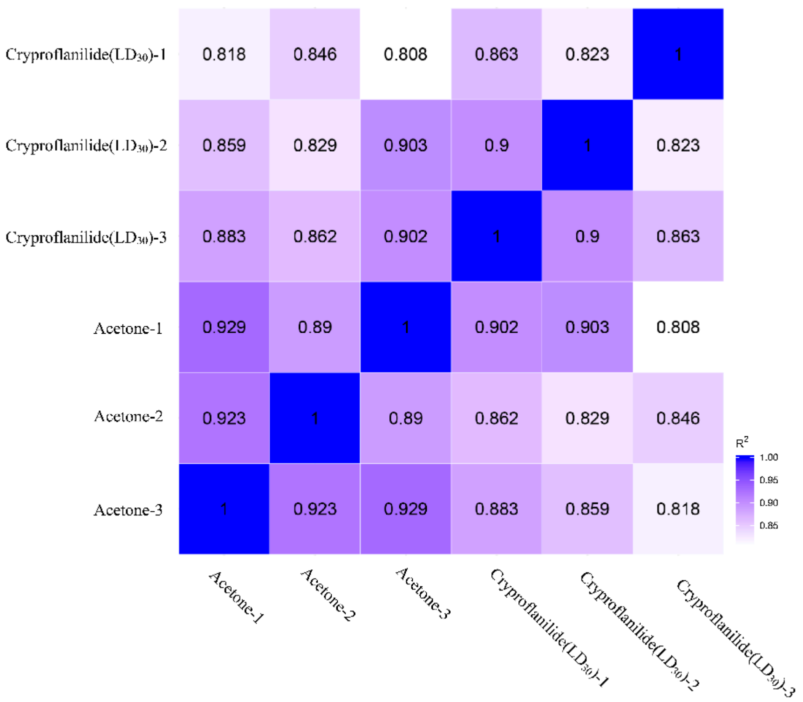

Figure S1 Between-sample correlation heatmap. The horizontal and vertical coordinates in the figure are the square of the correlation coefficients of each sample

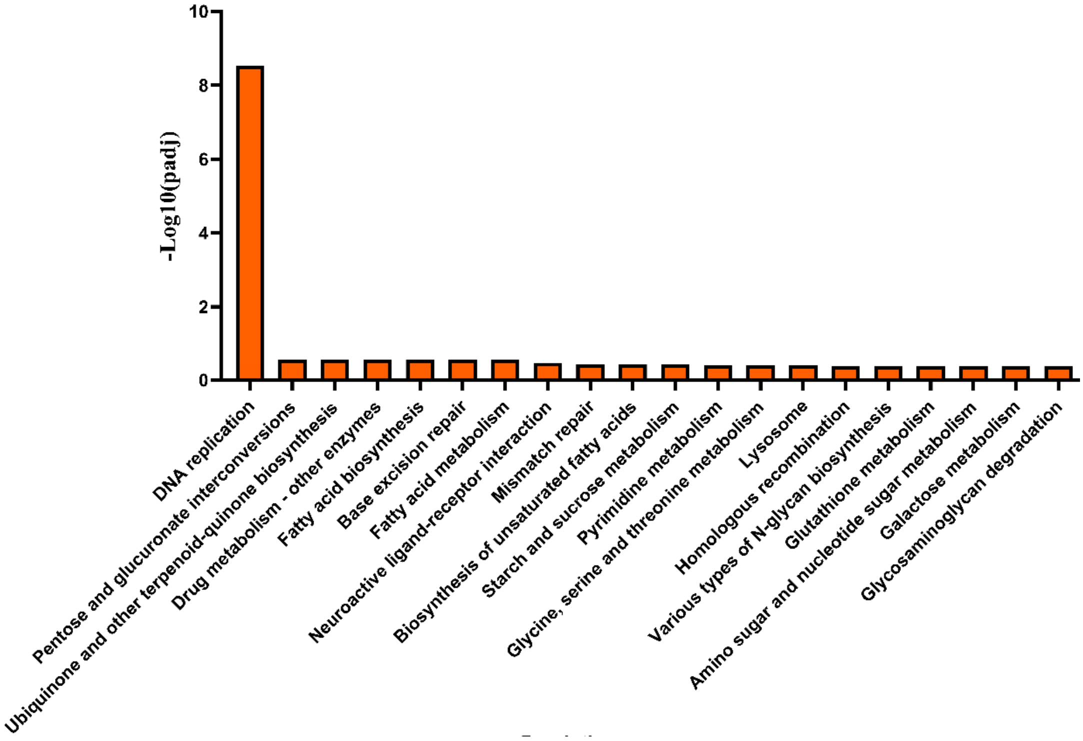

Figure S2 KEGG enrichment analysis histogram. The abscissa in the figure is the KEGG pathway, and the ordinate is the significance level of pathway enrichment.

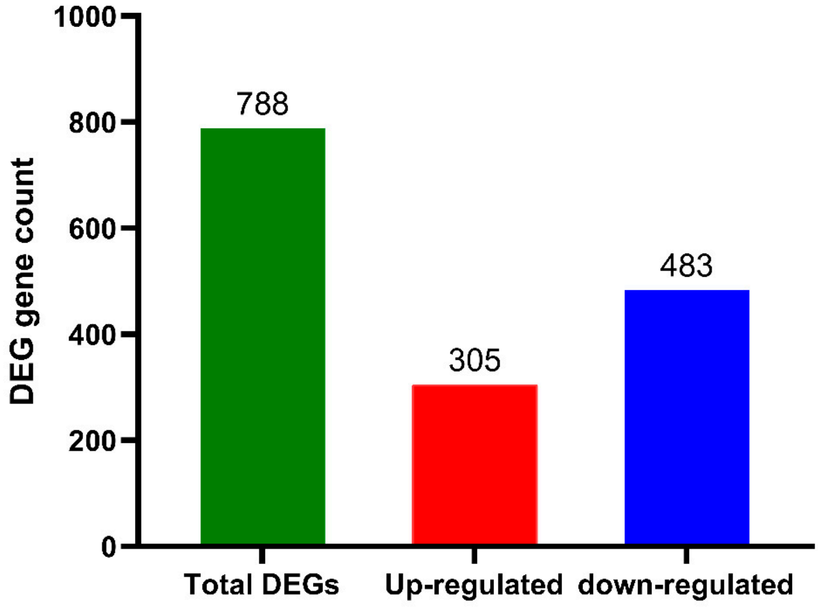

Figure S3 Difference gene counts.

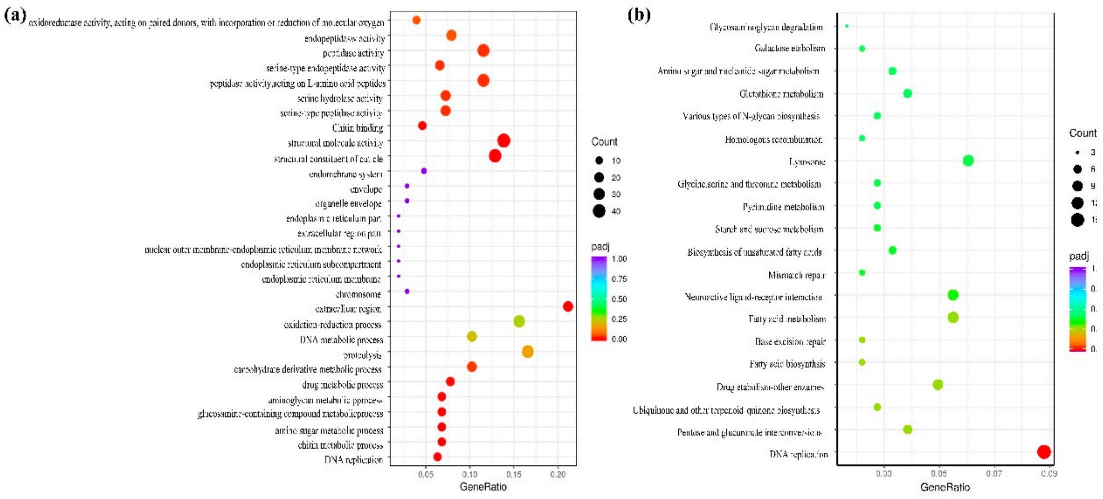

Figure S4 GO and KEGG enrichment pathways. (a): GO enrichment analysis scatter plot. The horizontal axis in the figure is the ratio of the number of differential genes annotated to the GO Term to the total number of differential genes, and the axis the GO Term. (b): KEGG enrichment analysis scatter plot. Ordinate indicates the KEGG pathways and the abscissa is the ratio of the number of differential genes annotated to KEGG pathway to the total number of differential gene.
